# Highly Efficient Conductivity Modulation via Stacked Multi-Gate Graphene Ambipolar Transistors

**DOI:** 10.3390/nano16030218

**Published:** 2026-02-06

**Authors:** Changbin Nie, Hongchen Zhang, Xianning Zhang, Feiying Sun, Jun Liu, Xingzhan Wei

**Affiliations:** 1 Chongqing Institute of Green and Intelligent Technology, Chinese Academy of Sciences, Chongqing 400714, China; niechangbin@cigit.ac.cn (C.N.); sunfeiying@cigit.ac.cn (F.S.); 2 University of Chinese Academy of Sciences, Beijing 100049, China; 3 Chongqing School, University of Chinese Academy of Sciences, Chongqing 400714, China; s230402034@stu.cqupt.edu.cn (H.Z.); zhangxianning@tju.edu.cn (X.Z.); 4 School of Optoelectronic Engineering, Chongqing University of Posts and Telecommunications, Chongqing 400065, China; 5 School of Optoelectronic Science and Engineering, University of Electronic Science and Technology of China, Chengdu 610054, China; 202312050839@std.uestc.edu.cn; 6 Hangzhou Hikmicro Sensing Technology, Hangzhou 310051, China

**Keywords:** vertical stacking, modulation, ambipolar transistor, graphene

## Abstract

The exceptional adjustability and ambipolar behavior of graphene offer significant potential for next-generation optoelectronics, where the conductivity of graphene is primarily modulated by the interface field of heterojunction. However, interface defects, which are inevitably introduced during fabrication, severely limit the effectiveness of gate voltage modulation. Although the layer-by-layer transfer method can effectively enhance conductivity, it also raises the carrier concentration and impairs the symmetry of ambipolar characteristics. This work presents a stacked multi-gate graphene transistor in which synergistic modulation enables efficient regulation of channel conductivity while maintaining low carrier concentration. Simulations are carried out to analyze how mobility, doping concentration, and the number of stacking layers influence the modulation of conductivity. Experimentally, a three-layer stacked graphene structure with distributed source and drain electrodes is fabricated. The device exhibits pronounced ambipolar transfer characteristics and demonstrates a clear improvement in transconductance compared to its conventional one-layer graphene counterpart. This research offers a feasible design strategy for high-performance, vertically integrated graphene-based electronic devices.

## 1. Introduction

The excellent carrier mobility of graphene and its unique ambipolar characteristics open vast prospects for the development of next-generation optoelectronic devices, such as photogating photodetectors [1,2,3,4,5] and brain-like sensors [6,7,8,9]. In such devices, the high transconductance directly determines the efficiency of gate voltage control over the channel current, making it a core parameter for improving photoelectric response. Moreover, the ambipolar characteristics enable the devices to generate balanced positive and negative photoelectric currents, which is crucial for simulating the excitatory and inhibitory signals. Therefore, possessing both high transconductance and symmetrical ambipolar characteristics is key to advancing graphene optoelectronic devices towards practical applications.

Currently, one primary strategy for realizing modulation involves constructing a planar heterojunction by combining monolayer graphene with traditional semiconductor materials [10,11,12,13,14]. In this configuration, photogenerated charge carriers are injected into the graphene channel through the built-in electric field at this interface, thereby modulating the graphene’s conductivity. However, interface defects and impurity scattering introduced during fabrication [15,16] prevent monolayer graphene from realizing its full potential, leading to suboptimal electrical modulation. While stacking multilayer graphene can enhance conductivity [17,18], it also substantially increases the intrinsic carrier concentration. This not only reduces gate control efficiency but also disrupts the inherent balance of ambipolar symmetry [19]. Furthermore, in planar heterojunctions, the limited electric field strength and narrow depletion region hinder both the effective separation and collection of photogenerated carriers, thereby ultimately limiting device performance [20].

In this paper, we propose a design strategy for vertically stacked graphene transistors based on multi-gate control. By implementing a stacked architecture, multiple gates synergistically modulate the electrical properties of the graphene channel. This approach maintains a low intrinsic doping concentration in the graphene while enhancing the modulation efficiency of the channel conductivity. Furthermore, the dielectric layer prevents doping of graphene during the fabrication process, ensuring good symmetry. We designed and fabricated a three-layer stacked graphene transistor and established a corresponding performance simulation model. The influence of graphene mobility, doping concentration, and layer count on modulation capability and transfer symmetry were analyzed. The devices exhibit excellent ambipolar characteristics. The transconductance improves threefold as stacking increases from one to three layers, with a concurrent Dirac point shift of 0.4 V. Moreover, the symmetry factor of current variation around the Dirac point exceeds 0.8, with a relative deviation below 0.2, confirming that the stacked structure enhances gate control efficiency while retaining favorable ambipolar symmetry. This work provides a feasible design approach for high-performance, vertically integrated multifunctional devices.

## 2. Materials and Methods

### 2.1. Device Simulation

The electrical properties of the devices were simulated using semiconductor physics simulation software (Synopsys, version 2018) [21,22,23]. The structural parameters, electrode positions, contact definitions, doping settings, and mesh generation were defined (Figure S1). Models such as Fermi-Dirac statistics, current continuity equation, Poisson’s equation, and Shockley-Read-Hall (SRH) recombination were introduced. The material parameters of graphene were modified based on the polycrystalline silicon material in the material library.

### 2.2. Device Fabrication

The fabrication process of the devices is schematically shown in Figure S2. First, LOR/S1805 photoresist was spin-coated onto a Si/SiO_2_ substrate, and the electrode pattern was defined using ultraviolet lithography. Cr/Au (5 nm/50 nm) was deposited onto the substrate surface using electron beam deposition, and the metal on the photoresist was removed using acetone and developer, followed by removal of the LOR layer to fabricate the source and drain electrodes. Subsequently, the monolayer graphene protected with PMMA was transferred onto the surface of the source and drain electrodes, and patterned into channels using UV exposure and oxygen plasma etching. Then, a 20 nm Al_2_O_3_ dielectric layer was deposited on the graphene surface using atomic layer deposition (ALD) at a temperature of 80 °C. A metal gate was fabricated on the dielectric layer surface using UV exposure, electron beam deposition, and lift-off process. An Al_2_O_3_ layer was deposited as an isolation layer using ALD, and contact holes were exposed and etched using UV lithography and wet etching processes, respectively. Finally, by repeatedly performing the above steps, monolayer graphene, source and drain electrodes, gate dielectric layer, and gate electrode were sequentially integrated to fabricate two-layer and three-layer stacked transistors. The microscopic characterization results at each process are shown in Figure S8. Furthermore, monolayer graphene was transferred and delaminated layer by layer using a PMMA-assisted method, resulting in two-layer and three-layer stacked graphene materials without Al_2_O_3_ for Raman spectroscopy testing.

### 2.3. Device Characterization and Testing

Raman spectroscopy (InVia Reflex, Renishaw, Gloucestershire, London) was used to characterize the graphene film by using a 532 nm laser with a power of 10 mW as the light source. The surface morphology of the devices was tested and characterized using an optical microscope and a scanning electron microscope (SEM). The IV and transfer characteristics of the devices were measured using a semiconductor parameter analyzer system (Keithley 4200-SCS, Keithley Instruments, Cleveland, OH, USA). The device electrodes were connected to the analyzer via an optical probe platform, and the schematic diagram of the device circuit is shown in Figure S10. For the measurement of the transistor IV characteristic curves, the source-drain voltage was swept from 0 V to 5 V with a step size of 20 mV, Range I was set to the 100 pA in the limited auto mode, and the Range V was set to the optimal correction mode. For the measurement of the transfer characteristic curves, the source-drain voltage was fixed at 0.1 V, and the gate voltage was scanned from −5 V to 5 V with a step size of 0.1 V. Range I and Range V were selected as the 100 mA and the “Best Fixed”, respectively.

## 3. Results

**Figure 1:**
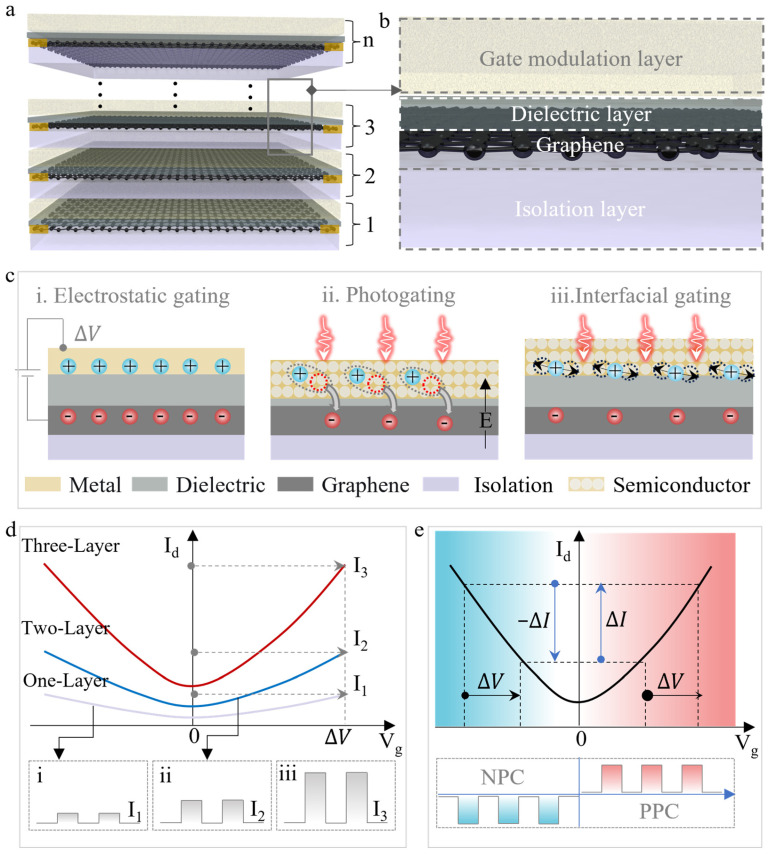
Schematic diagram of the vertically stacked graphene structure and its working principle. (**a**) Stacked graphene transistor structure; (**b**) Schematic diagram of the channel region in the stacked transistor; (**c**) Modulation mechanism of graphene under different gate modulation layers; (**d**) Enhancement effect of the vertically stacked structure on gate control; i–iii show schematic diagrams of the current change in one-layer, two-layer, and three-layer stacked devices, respectively, when the modulation voltage changes by ΔV. (**e**) Influence of ambipolar symmetry on positive and negative currents under the same modulation voltage.

Figure 1a schematically illustrates the vertically stacked graphene transistor. Each structural unit includes an isolation layer, a graphene layer, a dielectric layer, and a gate modulation layer, as shown in Figure 1b. The isolation layer is vertically located between two graphene transistor structures to prevent short circuit or breakdown during the gate modulation process. Graphene acts as the conductive channel for carrier transport and as the source and drain electrodes for current collection. The dielectric layer and the gate modulation layer work together to modulate the carrier concentration in the graphene channel.

The modulation mechanism of graphene channel conductivity is highly dependent on the gate material and dielectric thickness. Figure 1c shows three typical conductivity modulation methods, namely electrostatic gating [24,25], photogating [2,26], and interfacial gating [27,28]. When a metal gate is used, an applied bias accumulates charges at the gate/dielectric interface. Through the capacitive coupling effect of the dielectric layer, corresponding electrons or holes are induced in graphene, thus changing the channel conductivity, as shown in Figure 1c(i). When the metal gate is replaced with a semiconductor layer, the channel conductivity can be modulated by light. When the semiconductor layer is thin, photogenerated carriers can be directly injected into the graphene under the driving force of the built-in electric field at the interface, changing its current (Figure 1c(ii)). In contrast, when the semiconductor layer is thicker, photogenerated carriers are more easily trapped by interface trap states. These trapped charges act as a long-lasting gate voltage, inducing carriers in the graphene and thus modulating the channel current (Figure 1c(iii)).

By using multiple gates to modulate the conductivity of the graphene channel, the gate modulation capability is enhanced, allowing the graphene channel to achieve a larger current change under the same modulation voltage (Δ*V*), as shown in Figure 1d. Therefore, photodetectors based on photogating or interfacial gating mechanisms will exhibit larger photocurrents and responsivities in the stacked devices. Furthermore, due to the encapsulation and protection provided by the dielectric layer, external environmental doping of the graphene channel is avoided, resulting in a more symmetrical transfer characteristic curve. Under the same voltage change, the electron and hole portions generate positive photocurrents (PPC) and negative photocurrents (NPC) of nearly equal magnitude (Figure 1e), which is beneficial for improving the performance of synaptic-type devices.

**Figure 2:**
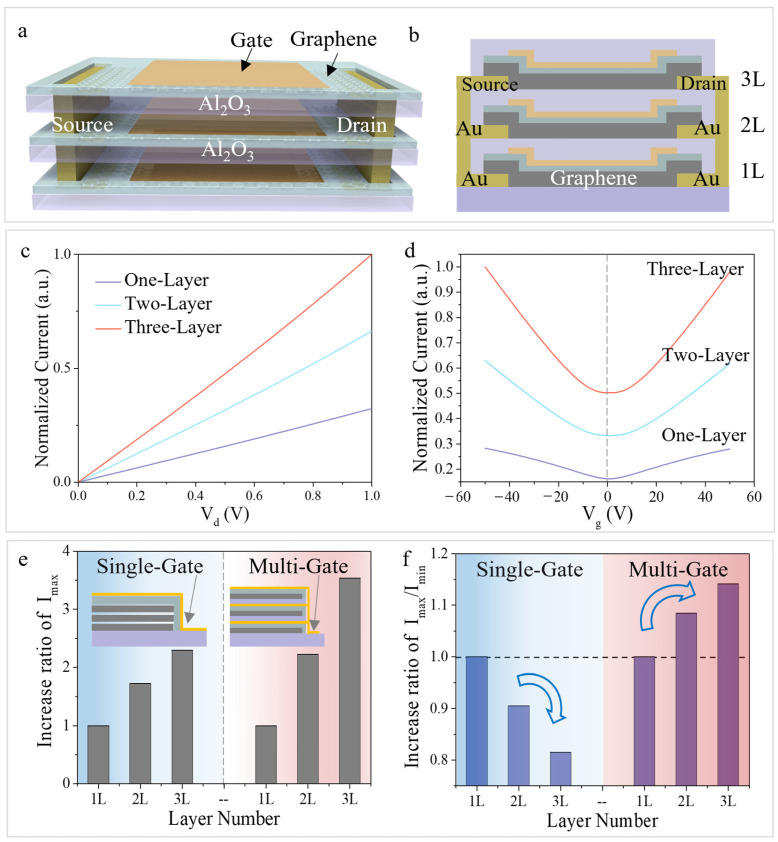
Electrical performance simulation of the three-layer stacked graphene transistor under metal gate modulation (**a**,**b**) Schematic diagram and cross-sectional view of the graphene transistor; (**c**) I-V curves of the graphene transistor with different stacking layers; (**d**) Transfer characteristic curves of the graphene transistor with different stacking layers; (**e**,**f**) Statistics of the increase ratios of *I_max_* and *I_max_*/*I_min_* in comparison with the one-layer graphene device, where *I_max_* and *I_min_* represent the maximum and minimum current values in the transfer curves (Figure S4), respectively.

To verify the modulation performance of the multi-gate structure on the graphene channel, simulation models of graphene transistors with different numbers of layers were established, and corresponding electrical properties were simulated (Figure S1). Figure 2a,b shows the three-dimensional structure and cross-sectional schematic diagram of the three-layer stacked transistor, respectively, which is realized by interconnecting the corresponding electrodes of all three layers.

To perform a qualitative analysis of the device, the I–V and transfer characteristic curves of transistors with different stacking layers were simulated, and the results are shown in Figure 2c,d. As the number of stacking layers increases, the device current increases obviously. From transfer curve, it can be observed that all devices exhibit typical ambipolar behavior. With the increase in the number of stacking layers, the peak current (*I_max_*) of the transfer curve increases markedly, and the current variation range (*I_max_*/*I_min_*) on both sides of the Dirac point also increases accordingly.

For the single-gate device, we performed simulation analysis on graphene transistors with different numbers of layers (Figure S3). Figure 2e,f statistically compares the I_max_ and current *I_max_*/*I_min_* of the transfer curves for devices under single-gate and multi-gate devices, respectively. The current of one-layer graphene transistor was normalized as a baseline. Simulations show that when the number of stacked layers increases from 1 to 3, the I_max_ of both structures effectively increases, demonstrating that the multi-gate structure exhibits a stronger current multiplication effect (Figure 2e). In terms of current modulation range, the *I_max_*/*I_min_* value of the single-gate device shows a decreasing trend with increasing layers, while the *I_max_*/*I_min_* of the multi-gate device increases with increasing layers (Figure 2f). This difference is mainly attributed to the different modulation mechanism of the two structures.

The multi-gate modulation structure essentially utilizes individual gates to independently modulate graphene layers, followed by summing the current of each layer. Single-gate modulation employs a single gate to modulate the three-layer stacked graphene, but its modulation capability is limited. Therefore, under multi-gate modulation, the three-layer stacked graphene transistor exhibits higher current multiplication capability and a larger current modulation amplitude.

Based on simulation models, the effects of carrier mobility, doping type, and doping concentration on transfer characteristics were studied. The results show that when the electron (or hole) mobility is kept constant while the mobility of the other carrier is reduced, the corresponding transconductance decreases, leading to a decrease in the ambipolar symmetry of the transfer curve (Figure S5). Furthermore, increasing the graphene doping concentration causes a remarkable shift in the Dirac point (Figure S6) and suppresses the gate modulation efficiency in the minority carrier region, thus degrading the symmetry of the transfer curve (Figure S7).

**Figure 3:**
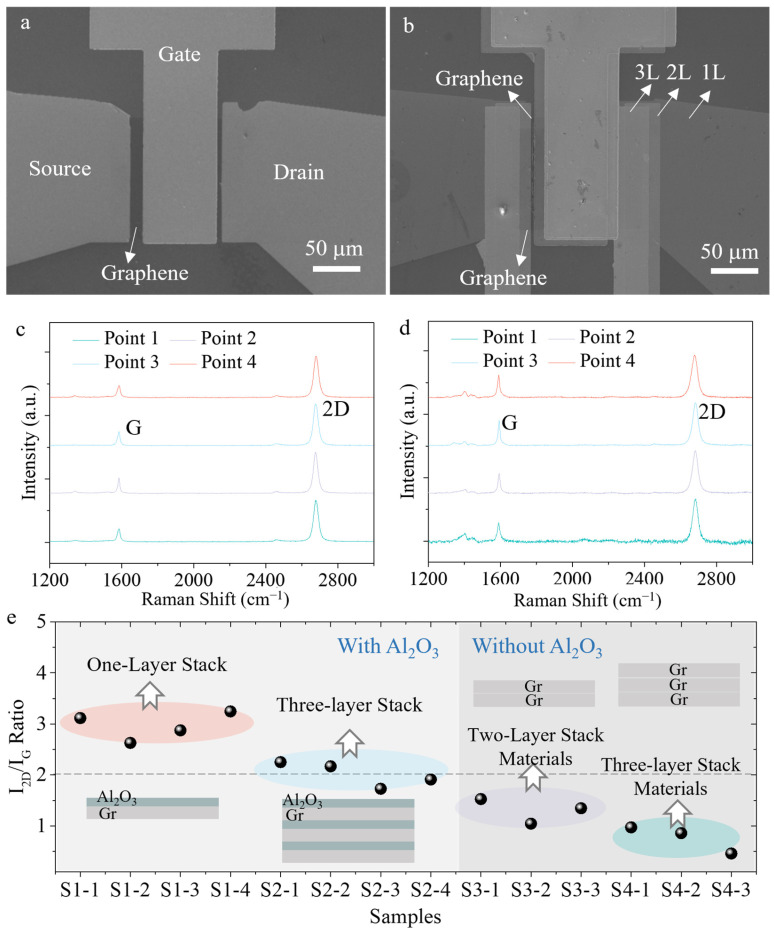
Characterization of the graphene transistors. (**a**) Microscope image of one-layer transistor; (**b**) Microscope image of three-layer stacked transistor; (**c**) Raman spectroscopy of one-layer transistor, with Points 1–4 indicating different locations on the device; (**d**) Raman spectroscopy of three-layer stacked transistor; (**e**) Statistical results of the intensity ratio of the Raman 2D peak to G peak for graphene with different structures, where S1 and S2 represent the one-layer and three-layers stacked graphene transistors with Al_2_O_3_ film, and S3 and S4 denote the two-layer and three-layer stacked graphene materials without Al_2_O_3_ film.

A low-temperature atomic layer deposition (ALD) process was developed to prepare Al_2_O_3_ dielectric layers. This process can passivate and protect the graphene channel interface, helping to maintain the symmetry of electron and hole mobility. Figure 3a,b shows SEM images of one-layer and three-layer stacked transistors, respectively. The SEM results indicate that the graphene channel maintains good cleanliness after the multi-step process, with no significant damage or wrinkles on the surface. Due to misalignment during exposure, there is a relative positional shift between the metal electrodes of each layer, which can be distinguished by the image contrast of different layers (Figure 3b). Furthermore, Raman spectroscopy characterization was performed on the graphene in both one-layer and three-layer stacked devices (Figure 3c,d). The Raman spectra of both devices show good uniformity, with the 2D peak intensity significantly higher than the G peak, which is consistent with typical single-layer graphene [29]. The D peak was almost undetectable in one-layer device, indicating that the fabrication process did not introduce obvious defects. Compared to the one-layer device, the D peak in the three-layer device is only slightly enhanced and remains much lower than the device without a dielectric protective layer (Figure S9), verifying that this fabrication process provides a favorable protection for the graphene film.

We performed statistical analysis on the intensity ratio of the 2D peak to the G peak in the Raman spectra (Figure 3e). It can be observed that the ratio of the one-layer device is approximately 3, while the ratio of the three-layer stacked device decreases slightly but remains around 2, indicating that the low-temperature Al_2_O_3_ deposition process results in less doping of the graphene film [30]. In contrast, the two-layer and three-layer stacked graphene materials without Al_2_O_3_ film exhibit a stepwise decrease, and the peak ratios are similar to the typical values for double-layer and triple-layer graphene [31]. These results demonstrate that the Al_2_O_3_ dielectric layer provides excellent isolation performance, allowing each monolayer graphene channel to be individually modulated by the gate.

**Figure 4:**
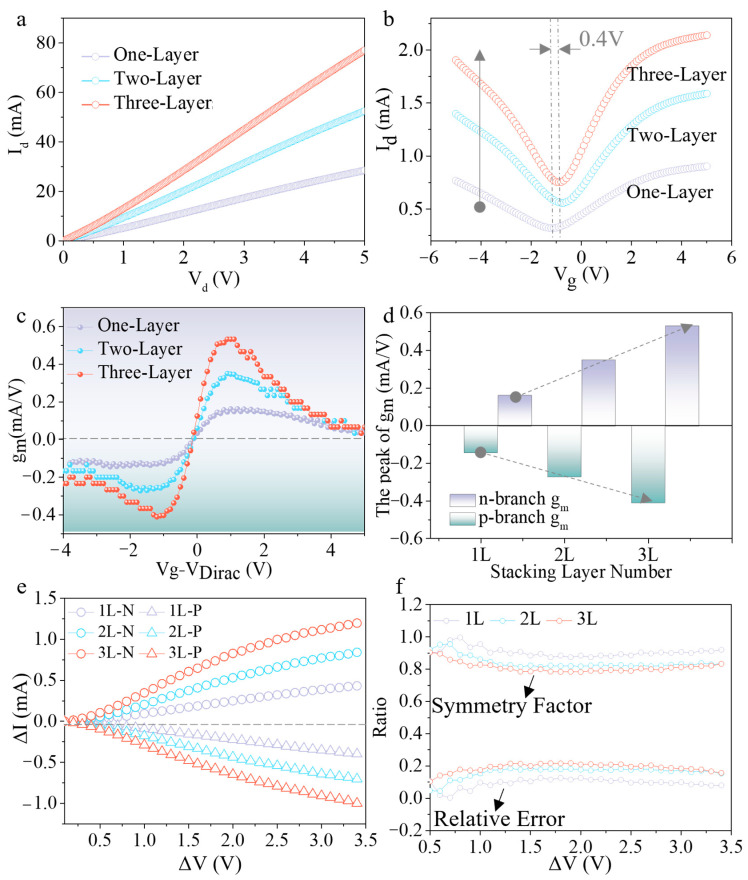
Experimental test results of electrical performance. (**a**) Transmission characteristics curves under different stacking layers; (**b**) Transfer characteristic curve changes with the number of stacked layers; (**c**) Relationship between calculated g_m_ of graphene and stacking layers, where the graphene width in the three structural devices is consistent; (**d**) Statistical results of *g_m_* peak values under different layers; (**e**) Currents in the n-type and p-type branches varies with Δ*V*, where Δ*V* represents the change in modulation voltage to the left (P-branch) and right (N-branch) relative to the Dirac point position. (**f**) Statistical results of symmetry factor and relative error of n-branch and p-branch current variations. The symmetry factor is calculated by the formula ($min\left\{\left|∆I_{e}\right|,\left|{∆I}_{h}\right|\right\}/max\left\{\left|∆I_{e}\right|,\left|∆I_{h}\right|\right\}$). The relative error is determined by the formula ($\left|∆I_{e}-∆I_{h}\right|/\left|{∆I}_{h}\right|$), where $∆I_{e}$ and $∆I_{h}$ represent the current changes (Δ*I*) of the n-branch and p-branch corresponding to the applied voltage change (Δ*V*), respectively.

Electrical measurements were performed on the fabricated devices, and Figure 4a shows the relevant characteristic curves. All devices exhibit good ohmic contact behaviors, and the current increase distinctly with the number of stacked layers. Figure 4b shows the transfer characteristic curves where the channel current increase with the number of stacked layers. The Dirac point shifts approximately 0.4 V, indicating good control of the doping level during the stacking process. Subsequently, the transconductance (*g_m_*) of the devices was calculated, and the results are shown in Figure 4c. On the left side of the Dirac point (hole branch) and the right side (electron branch), *g_m_* shows negative and positive values, respectively. The shape of the two branches as a function of gate voltage is basically symmetrical, revealing that the device has favorable ambipolar transport symmetry. Figure 4d shows the statistical results of the *g_m_* peaks for the electron branch and hole branch. The *g_m_* peak shows a linear increasing trend with the increase in the number of stacked layers. Compared with the one-layer device, the *g_m_* of the three-layer transistor increases by nearly 3 times, which verifies the enhanced current modulation capability of the stacked devices.

Furthermore, the current change (Δ*I*) on both sides of the Dirac point under different gate voltage variations (Δ*V*) was calculated, as shown in Figure 4e. Δ*I* is negative for the hole branch and positive for the electron branch, consistent with the polarity of the transconductance, and the absolute value of Δ*I* increases with the number of stacked layers. Subsequently, the symmetry factor and relative deviation of Δ*I* for different branches were calculated, as shown in Figure 4f. The symmetry factor of all devices is higher than 0.8, and the relative deviation is lower than 0.2, indicating that the devices exhibit excellent ambipolar symmetry. It is worth noting that the symmetry decreases slightly with increasing number of layers, which is due to the cumulative effect of intrinsic doping in graphene.

## 4. Discussion

This work extends the traditional graphene transistor structure in a vertical dimension. Each channel layer of the vertically stacked graphene transistor can be independently modulated by the corresponding gate, thus achieving a high modulation efficiency while maintaining a low carrier concentration. When high-performance top gate structures [32,33] or double gate structures [34,35] are vertically stacked, it can further improve their modulation efficiency and overall device performance. Moreover, the low-temperature ALD process utilized can effectively avoid the degradation of transfer curve symmetry induced by defect generation or unintended doping. Compared with the previously reported stacked graphene transistors [36,37], the devices fabricated in this work combine low Dirac point offset, high gate controllability, and bipolar symmetry (Table S2).

We have experimentally and numerically verified the enhanced gate modulation capability through multi-layer stacked structure for the graphene channel. The quantitative analysis of the hysteresis effect will help further optimize the dielectric layer deposition process [38,39]. Furthermore, improving the quality of graphene [40], optimizing material transfer processes [41], or increasing the number of stacked layers can further enhance device performance. This scheme offers a high degree of freedom in structural design. For instance, multi-wavelength photodetection can be realized by replacing the gate layers with semiconductor materials of different bandgaps [42,43]. The construction of intelligent devices capable of multimodal parallel processing can be enabled by employing ferroelectric as the gate layers to emulate synaptic weights [44,45].

## 5. Conclusions

This work introduces a multi-gate ambipolar transistor architecture based on vertically stacked graphene, which systematically investigates the modulation mechanism of the gate dielectric layer on graphene channel conductivity. Through modelling the electrical properties under gate control, the enhanced modulation efficiency of the stacked devices has been verified. A low-temperature dielectric layer deposition process has been developed to avoid the influence of defects and doping on the symmetry of the transfer curve. The three-layer stacked device achieves an obvious improvement in transconductance, nearly 3 times that of a one-layer device, while maintaining a low doping concentration (Dirac point shift of only 0.4 V) and high symmetry (symmetry factor > 0.8). This research provides a scalable vertical integration approach for high-performance, programmable graphene-based optoelectronics.

## Data Availability

All data needed to evaluate the conclusions in the paper are present in the paper and Supplementary Materials. The relevant data can be obtained from the corresponding author after obtaining permission.

## Supplementary Materials

The following supporting information can be downloaded at: https://www.mdpi.com/article/10.3390/nano16030218/s1, Table S1: Graphene parameters used in the simulation. Figure S1: Graphene transistor simulation modeling. (a) Cross-sectional diagram of the device; (b) Device simulation modeling process; (c) Structural mesh definition. Figure S2: Process flow diagram for the fabrication of one-layer graphene transistors. Figure S3. Schematic diagram of graphene transistor and device simulation. (a) Structure of three-layer graphene transistor with single-gate control; (b,c) I-V_d_ curves and transfer characteristics of graphene transistors with different numbers of layers under single-gate control. Figure S4. Simulation current values of single gate modulation and multi gate modulation transistors. Figure S5. Simulated electrical performance of graphene devices under different mobilities. (a,b) I-V_d_ curves and transfer characteristics curves of the devices under different electron mobilities; (c,d) I-V_d_ curves and transfer characteristics curves of the devices under different hole mobilities. Figure S6. Simulation of the effect of graphene doping type on the position of the graphene Dirac point. (a) N-type doping. The graphene Dirac point is on the negative gate voltage side; (b) P-type doping. The graphene Dirac point is on the positive gate voltage side. Figure S7. Simulation of the electrical properties of graphene devices at different doping concentrations. (a) Transfer curves of graphene devices at different P-type doping concentrations; (b) Transfer curves of graphene devices at different N-type doping concentrations; (c) Statistical results of the Dirac point position at different doping concentrations. Figure S8. Microscopic characterization. (a,b) One-layer graphene device; (c,d) Single gate-driven graphene device; (e) Two-layer stacked device; (f) Three-layer stacked device. Figure S9. Raman characterization of graphene. (a) Raman characterization results at different locations on the two-layer stacked graphene without Al_2_O_3_ film; (b) Raman characterization results at different points on the three-layer stacked graphene without Al_2_O_3_ film. Figure S10. Schematic diagrams of transistor devices and relevant circuits. (a,c,e) represent top views of one-layer, two-layer, and three-layer stacked transistor devices, respectively; (b,d,f) show circuit diagrams of corresponding transistors. Figure S11. Ig–Vg curve of the graphene transistors with Al_2_O_3_ layer. Table S2. Performance comparison of multilayer stacked graphene transistors.
