# Supplementary material for: Highly Efficient Conductivity Modulation via Stacked Multi-Gate Graphene Ambipolar Transistors
Source: Nanomaterials (Basel). 2026 Feb 6;16(3):218. doi: 10.3390/nano16030218 (PMC12899657; doi:10.3390/nano16030218)
Supplement: Supplementary file 1 [file nanomaterials-16-00218-s001.zip › nanomaterials-4102837-supplementary.pdf]

Supporting Information

Highly Efficient Conductivity Modulation via Stacked Multi-Gate Graphene Ambipolar Transistors

Changbin Nie[1,2], Hongchen Zhang[3,5], Xianning Zhang[3,5], Feiying Sun[1,2], Jun Liu[4,6] and Xingzhan Wei[1,2,3*]

1 Chongqing Institute of Green and Intelligent Technology, Chinese Academy of Sciences, Chongqing 400714, China

2 University of Chinese Academy of Sciences, Beijing 100049, China

3 Chongqing School, University of Chinese Academy of Sciences, Chongqing 400714, China

4 School of Optoelectronic Science and Engineering, University of Electronic Science and Technology of China, Chengdu 610054, China

5 School of Optoelectronic Engineering, Chongqing University of Posts and Telecommunications, Chongqing 400065, China

6 Hangzhou Hikmicro Sensing Technology, Hangzhou, 310000, China

*Corresponding author E-mails: weixingzhan@cigit.ac.cn

The electrical characteristics of the device were simulated using TCAD software with models including Fermi-Dirac statistics, current continuity equation, Poisson's equation, and Shockley-Read-Hall (SRH) recombination model. Among these, the Fermi-Dirac statistics model is used to depict the carrier distribution under high doping or high carrier concentration conditions. In comparison with the traditional Boltzmann approximation, Fermi-Dirac statistics can more accurately describe the carrier behavior in degenerate semiconductors. The concentrations of electrons and holes are determined by the following equations:

$$n = N_C F_{1/2}\left(\frac{E_{F,n}-E_C}{kT}\right) \tag{S1}$$

$$p = N_V F_{1/2}\left(\frac{E_V-E_{F,p}}{kT}\right) \tag{S2}$$

where N_C and N_V denote the effective density of states in the conduction band and valence band, respectively, $E_{F,n}$ and $E_{F,p}$ represent the quasi-Fermi levels for electrons and holes, and $F_{1/2}$ is the Fermi-Dirac integral function of order 1/2.

The current continuity equation describes the spatiotemporal evolution of carrier concentration, incorporating the mechanisms of drift, diffusion, generation and recombination.

Electron continuity equation:

$$\frac{\partial n}{\partial t} = \frac{1}{q}\nabla \cdot \mathrm{J}_n + G_n - R_n \tag{S3}$$

Hole continuity equation:

$$\frac{\partial p}{\partial t} = -\frac{1}{q}\nabla \cdot \mathrm{J}_p + G_p - R_p \tag{S4}$$

where J_n and J_p are the electron and hole current densities, while G and R represent the carrier generation and recombination rates, respectively. The current density is given by the drift-diffusion model.

$$\mathrm{J}_n = q\mu_n n \nabla \Phi_n + qD_n \nabla n \tag{S5}$$

$$\mathrm{J}_p = q\mu_p p \nabla \Phi_p - qD_p \nabla p \tag{S6}$$

Poisson's equation correlates the electrostatic potential with the space charge distribution, and it is one of the fundamental equations for device simulation:

$$\nabla \cdot (\varepsilon \nabla \phi) = -q\left(p - n + N_D^+ - N_A^- + \rho_{trap}\right) \tag{S7}$$

ϕ denotes the electrostatic potential, ε the permittivity, N_D^+ and N_A^- represent the donor and acceptor concentrations, respectively, and ρ_{trap} is the trap charge density.

The Shockley-Read-Hall (SRH) recombination model describes the non-radiative recombination process via deep-level defects, and it is one of the dominant carrier loss mechanisms in semiconductor devices:

$$R_{SRH} = \frac{np - n_{i,eff}^2}{\tau_p(n+n_1) + \tau_n(p+p_1)} \tag{S8}$$

where $n_{i,eff}$ is the effective intrinsic carrier concentration (cm^{-3}), τ_n and τ_p denote the electron lifetime and hole lifetime (s), respectively, and n_1 and p_1 represent the electron and hole concentrations at the trap energy level (cm^{-3}).

The material parameters of graphene were modified based on those of polycrystalline silicon in the software material library. The graphene material parameters adopted in this work

are listed in Table 1.

Table S1. Graphene parameters used in the simulation.

Description	Unit	Value	Ref
Thickness	nm	5	-
Band gap	eV	0 eV	[1]
Relative permittivity		6	[1]
Optical constant	N/A	(n & k value)	[2]
Hole mobility	$cm^2V^{-1}s^{-1}$	10^4	[3]
Electron mobility	$cm^2V^{-1}s^{-1}$	10^4	[3]
Intrinsic carrier concentration	cm^{-2}	1×10^{12}	[3]
Electron affinity	eV	4.248 eV	[4]

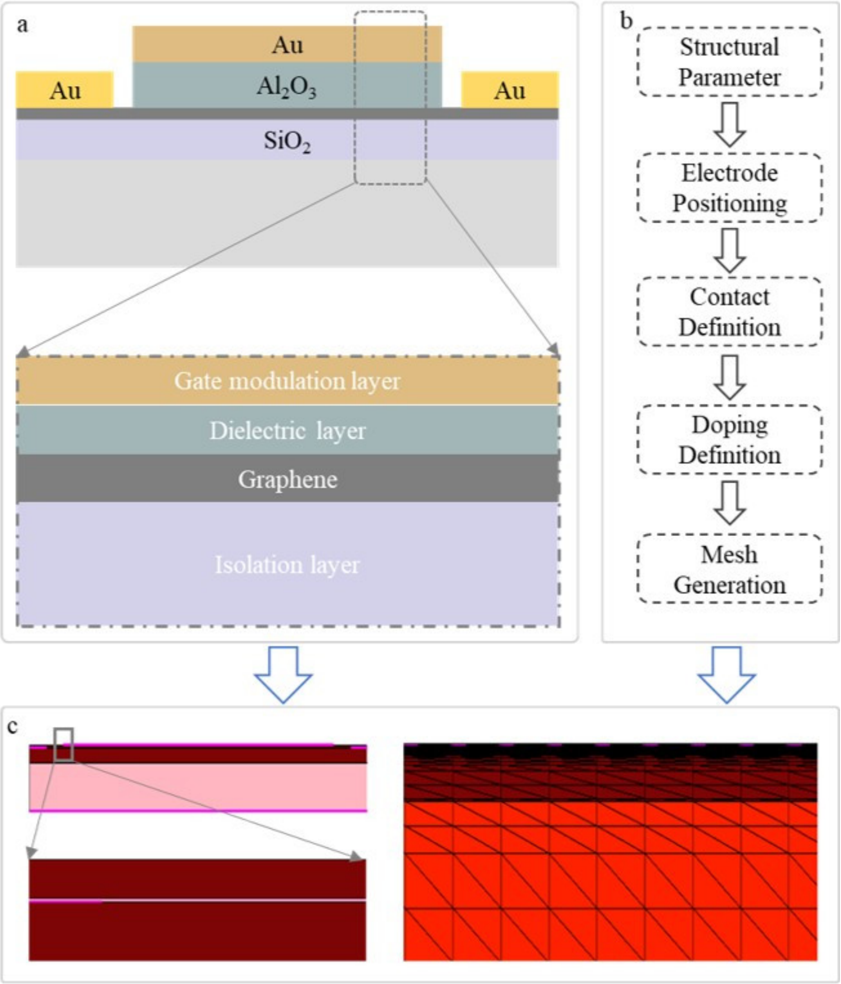

Figure S1. Graphene transistor simulation modeling. **(a)** Cross-sectional diagram of the device; **(b)** Device simulation modeling process; **(c)** Structural mesh definition.

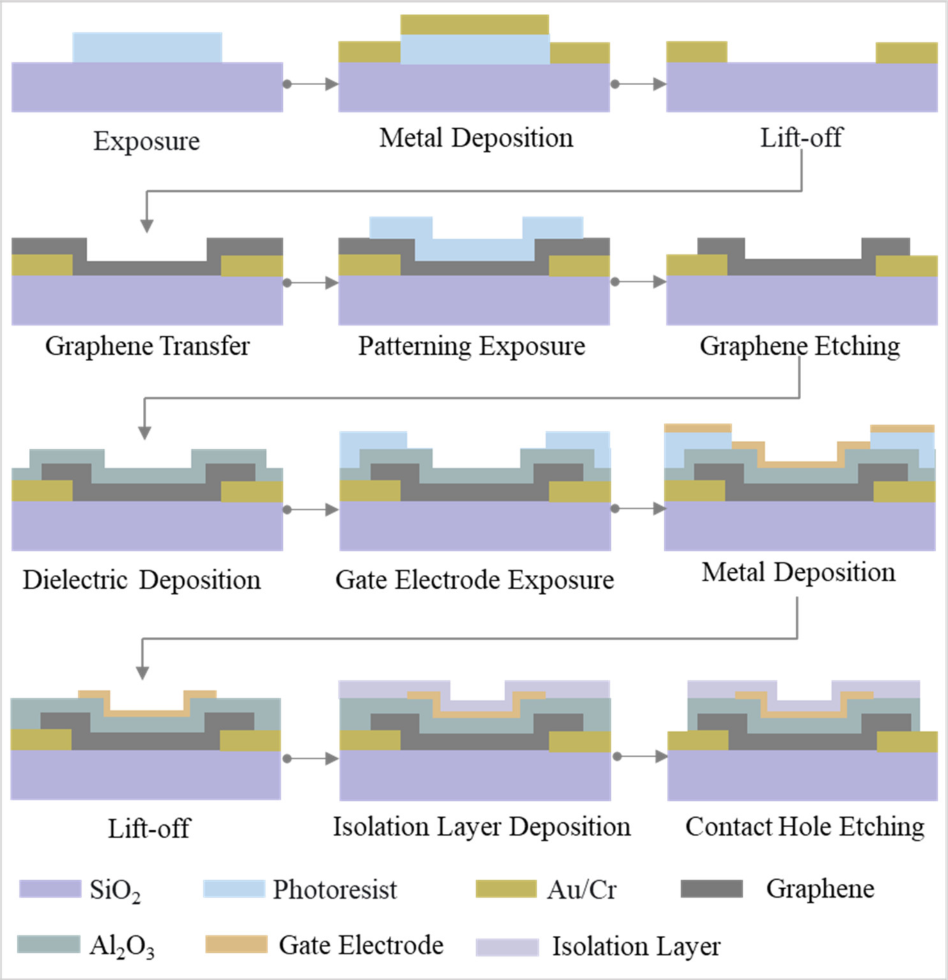

Figure S2. Process flow diagram for the fabrication of one-layer graphene transistors.

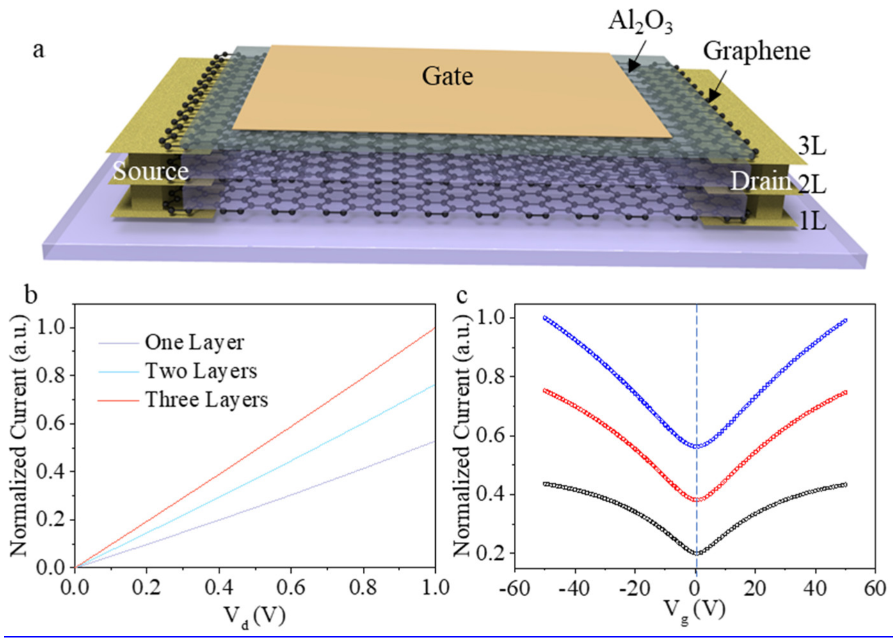

Figure S3. Schematic diagram of graphene transistor and device simulation. **(a)** Structure of three-layer graphene transistor with single-gate control; **(b, c)** I-V_d curves and transfer characteristics of graphene transistors with different numbers of layers under single-gate control.

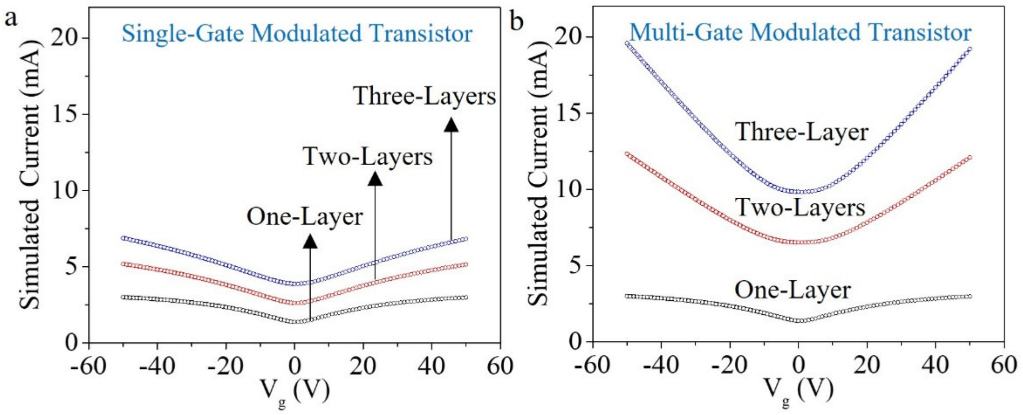

Figure S4. Simulation current values of single gate modulation and multi gate modulation transistors.

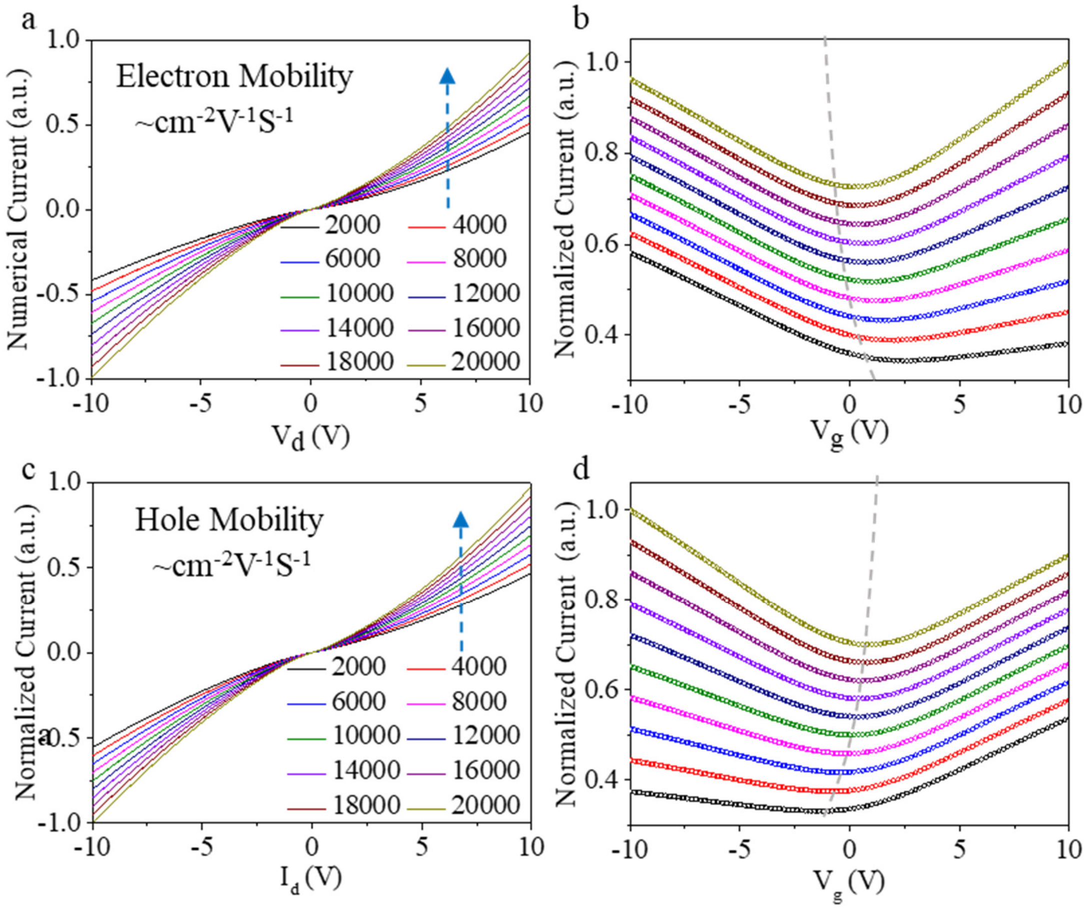

Figure S5. Simulated electrical performance of graphene devices under different mobilities. **(a, b)** I-V_d curves and transfer characteristics curves of the devices under different electron mobilities; **(c, d)** I-V_d curves and transfer characteristics curves of the devices under different hole mobilities.

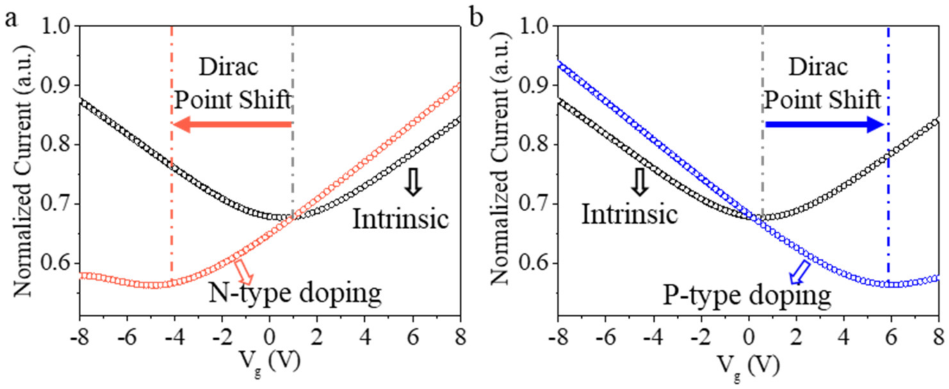

Figure S6. Simulation of the effect of graphene doping type on the position of the graphene Dirac point. **(a)** N-type doping. The graphene Dirac point is on the negative gate voltage side; **(b)** P-type doping. The graphene Dirac point is on the positive gate voltage side.

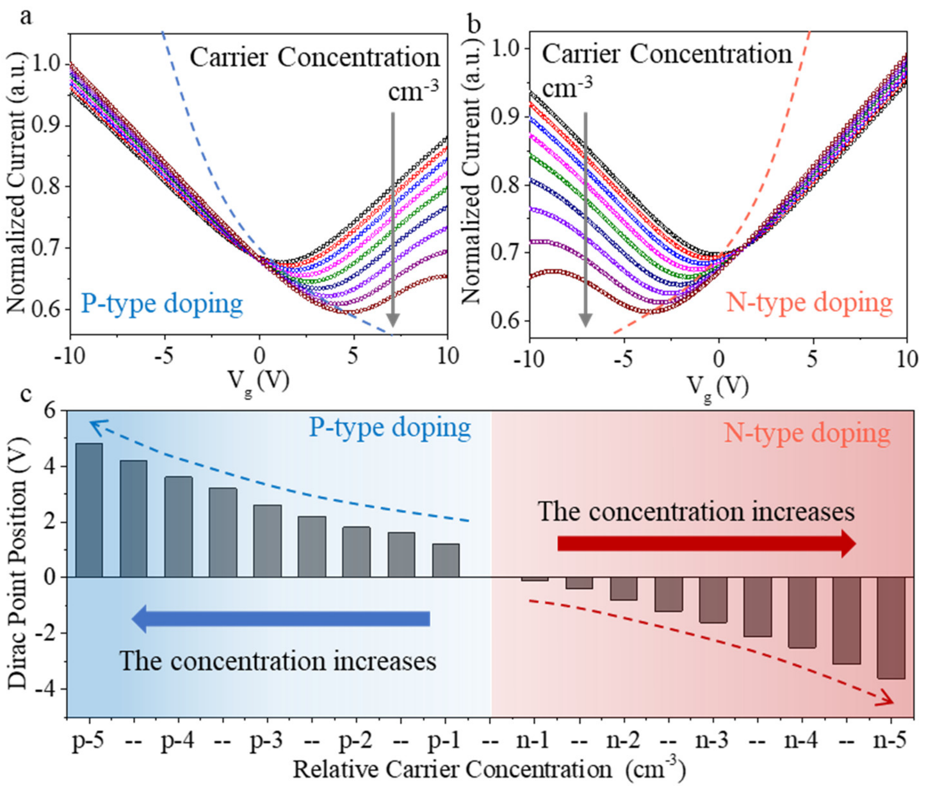

Figure S7. Simulation of the electrical properties of graphene devices at different doping concentrations. **(a)** Transfer curves of graphene devices at different P-type doping concentrations; **(b)** Transfer curves of graphene devices at different N-type doping concentrations; **(c)** Statistical results of the Dirac point position at different doping concentrations.

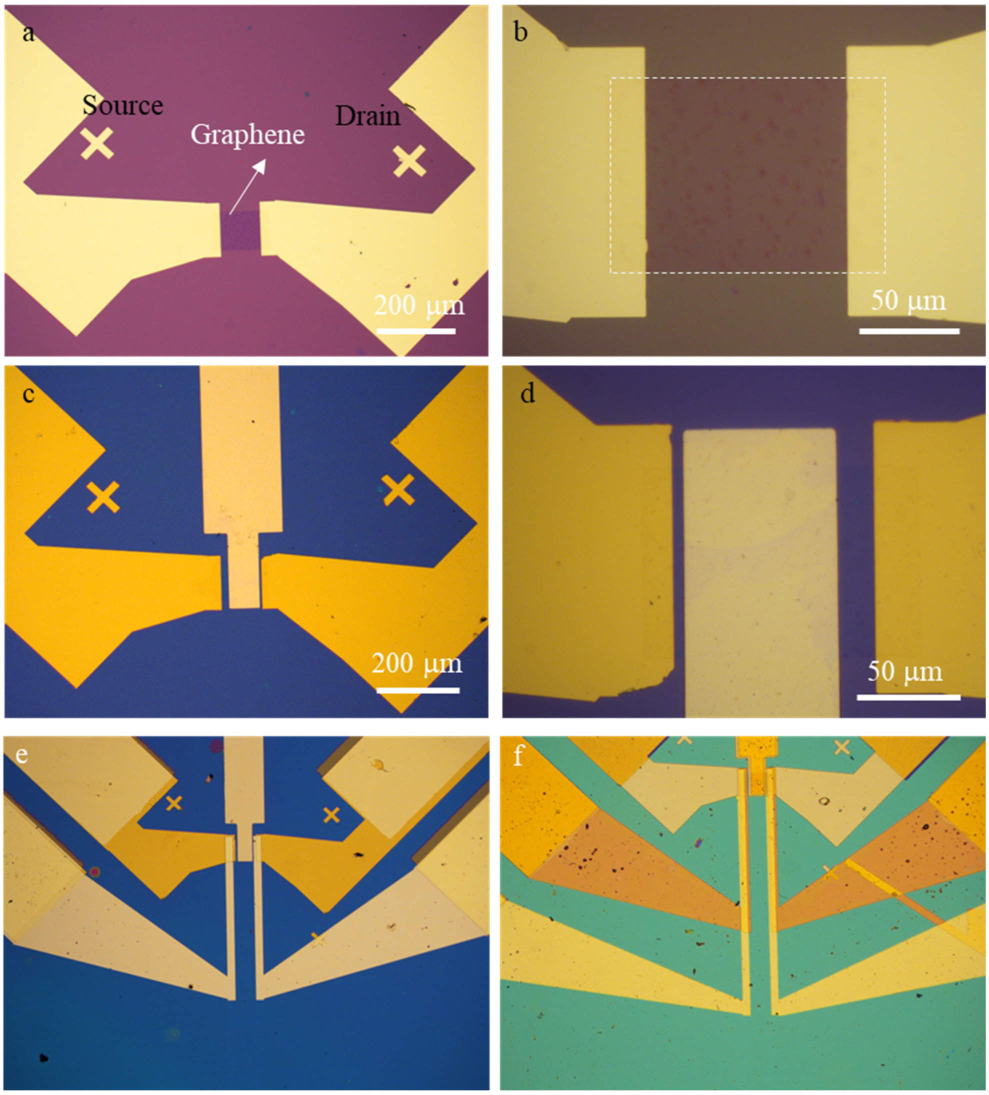

Figure S8. Microscopic characterization. **(a, b)** One-layer graphene device; **(c, d)** Single gate-driven graphene device; **(e)** Two-layer stacked device; **(f)** Three-layer stacked device.

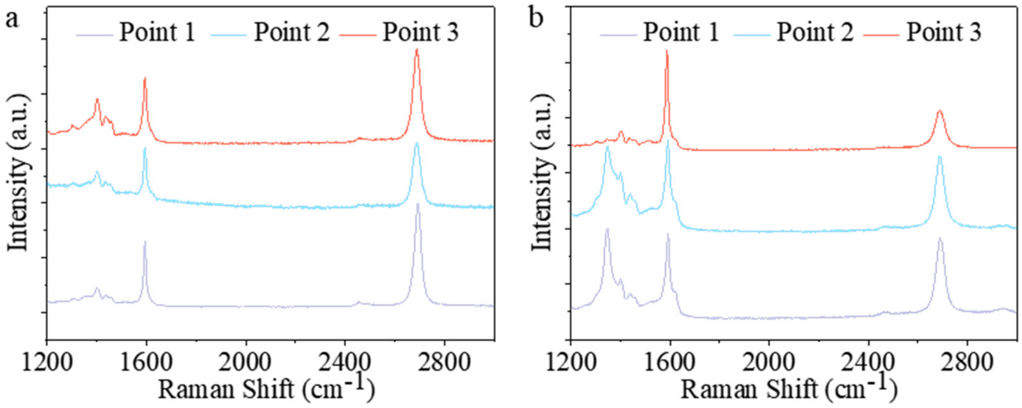

Figure S9. Raman characterization of graphene. (a) Raman characterization results at different locations on the two-layer stacked graphene without Al_2O_3 film; (b) Raman characterization results at different points on the three-layer stacked graphene without Al_2O_3 film.

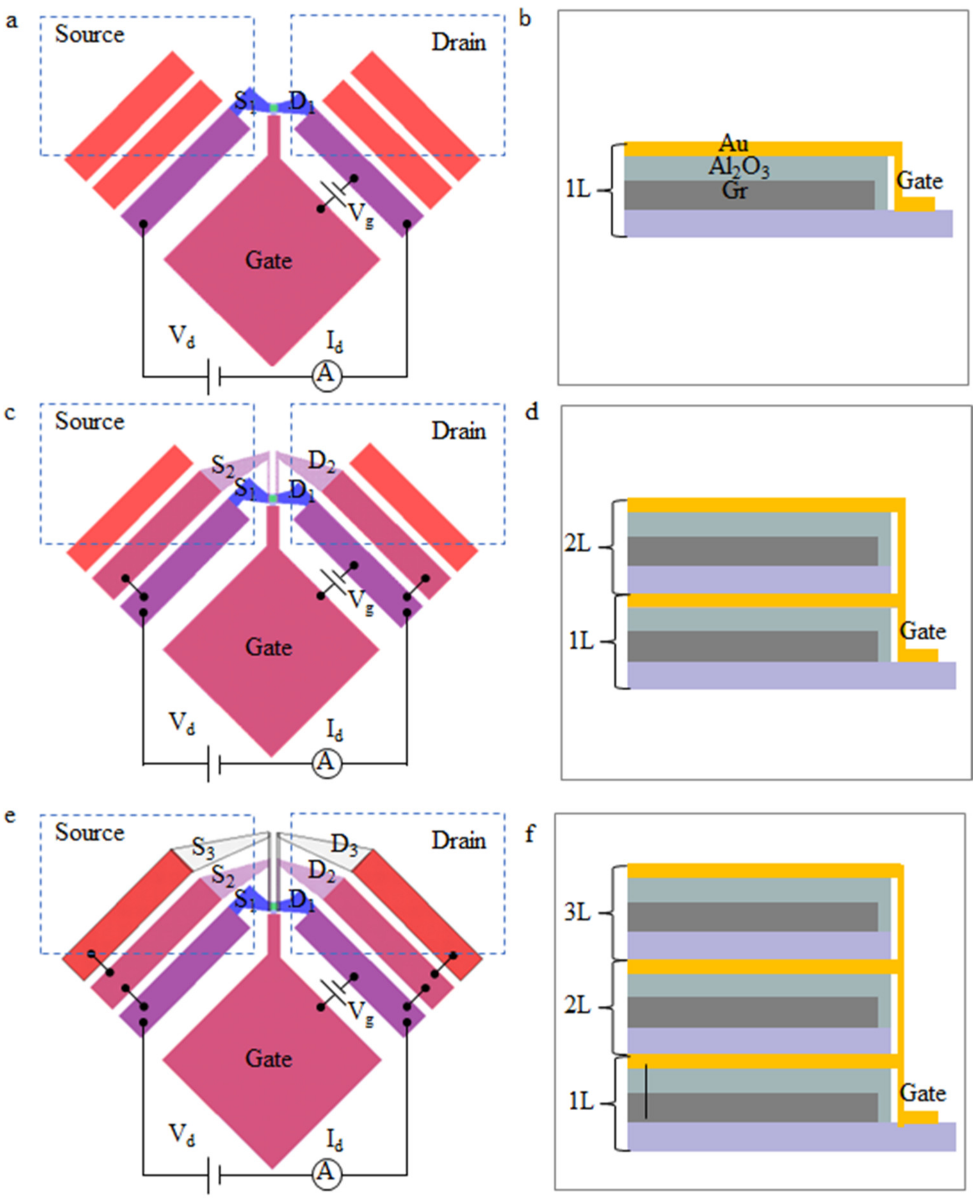

Figure S10. Schematic diagrams of transistor devices and relevant circuits. (a), (c), and (e) represent top views of one-layer, two-layer, and three-layer stacked transistor devices, respectively; (b), (d), and (f) show circuit diagrams of corresponding transistors.

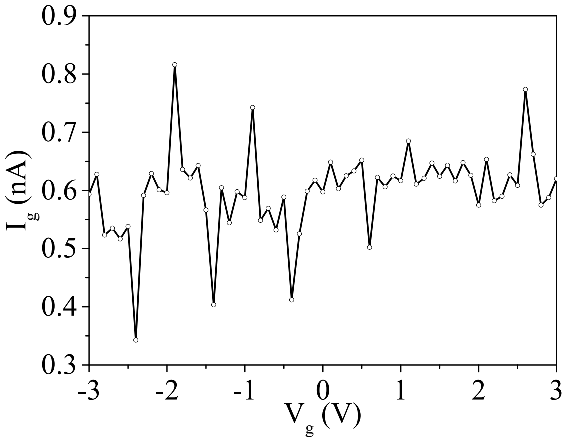

Figure S11. Ig–Vg curve of the graphene transistors with Al_2O_3 layer.

Table S2. Performance comparison of multilayer stacked graphene transistors.

Interlayer structure	Dielectric	Number of Stacked	Dirac Point Position	Trend with the number of layers	Symmetry	Ref.
without dielectric	TEOS-SiO_2	1-3 layers	~28 V@1L<br>~22 V@2L<br>~30 V@3L	nonlinear	Bipolar symmetric	[5]
with dielectric	HfO_2	1-3 layers	0 V @1L<br>0.5 V @2L<br>1 V @3L	Linear	N-branch suppressed	[6]
with dielectric	Al_2O_3	1-3 layers	-1.2 V @1L<br>-0.8 V @2L<br>-0.9 V @3L	Linear	Bipolar symmetric	This work

References

1. Fu J, Jiang H, Nie C, et al. Polarity-tunable field effect phototransistors[J]. Nano Letters, 2023, 23(11): 4923-4930.

2.Nair, R. R.; Blake, P.; Grigorenko, A. N.; Novoselov, K. S.; Geim, A. K. Optical Constants of Graphene Measured by Spectroscopic Ellipsometry. *Appl. Phys. Lett*. **2008**, 92, 183104.

3. Liu, Z.; Chen, J.; Wang, L.; et al. Schottky Infrared Detectors with Optically Tunable Barrier Beyond the Internal Photoemission Limit. *The Innovation.* **2023**, 4, 100389.

4. Zhang, C.; Liu, Y.; Li, W.; et al. Enhanced Photogating Effect in Graphene Photodetectors via Potential Fluctuation Engineering. *ACS Nano*. **2019**, 13, 11245–11254.

5. Shimatani M, Ikuta T, Sakamoto Y, et al. Turbostratic stacked graphene-based high-responsivity mid-wavelength infrared detector using an enhanced photogating effect[J]. Optical Materials Express, 2022, 12(2): 458-467.

6. Franklin A D, Oida S, Farmer D B, et al. Stacking Graphene Channels in Parallel for Enhanced Performance With the Same Footprint[J]. IEEE electron device letters, 2013, 34(4): 556-558.
